# Gut microbiota: A new therapeutic target for diabetic cardiomyopathy

**DOI:** 10.3389/fphar.2022.963672

**Published:** 2022-08-26

**Authors:** Suxin Yuan, Zhengyao Cai, Xingzhao Luan, Haibo Wang, Yi Zhong, Li Deng, Jian Feng

**Affiliations:** ^1^ Department of Cardiology, The Affiliated Hospital of Southwest Medical University, Luzhou, Sichuan, China; ^2^ Department of Neurosurgery, The Affiliated Hospital of Southwest Medical University, Luzhou, Sichuan, China; ^3^ Department of Cardiology, Gulin People’s Hospital, Luzhou, Sichuan, China; ^4^ Department of Rheumatology, The Affiliated, Hospital of Southwest Medical University, Luzhou, Sichaun, China

**Keywords:** diabetic cardiomyopathy, gut microbiota, oxidative stress, inflammation, apoptosis, autophagy

## Abstract

Diabetic cardiomyopathy seriously affects quality of life and even threatens life safety of patients. The pathogenesis of diabetic cardiomyopathy is complex and multifactorial, and it is widely accepted that its mechanisms include oxidative stress, inflammation, insulin resistance, apoptosis, and autophagy. Some studies have shown that gut microbiota plays an important role in cardiovascular diseases. Gut microbiota and its metabolites can affect the development of diabetic cardiomyopathy by regulating oxidative stress, inflammation, insulin resistance, apoptosis, and autophagy. Here, the mechanisms of gut microbiota and its metabolites resulting in diabetic cardiomyopathy are reviewed. Gut microbiota may be a new therapeutic target for diabetic cardiomyopathy.

## Introduction

Diabetic cardiomyopathy (DCM) refers to the existence of abnormal myocardial structure and performance in individuals with diabetes mellitus (DM) in the absence of other cardiac risk factors such as hypertension, coronary artery disease and significant valvular disease (Jia et al., 2018a). DCM is a pathophysiological condition that is associated with DM and can lead to heart failure (Dillmann, 2019), which is initially characterized by remodeling, myocardial fibrosis, and associated diastolic dysfunction, which is followed by systolic dysfunction, and ultimately by clinical heart failure (Jia et al., 2018a). The pathophysiological factors in patients with diabetes that drive the development of cardiomyopathy include oxidative stress (Tang et al., 2019), insulin resistance, inflammation (Jia et al., 2018a), autophagy (Dewanjee et al., 2021), cell apoptosis (Zhang et al., 2016), and pyroptosis (Shi et al., 2021). Cardiovascular diseases (CVD) are the leading cause of death with DCM among diabetes mellitus regardless previous risks for coronary disease, and the CVD risk of cardiomyopathy is 2–5 times higher than in non-diabetic patients (Kannel et al., 1974). Therefore, it is highly important to find a new target for the treatment of DCM.

Gut microbiota creates a unique ecosystem. It is considered an endocrine organ (Brown and Hazen, 2015). Recent studies have demonstrated that gut microbiota plays a significant role in human health and in diseases such as CVD, atherosclerosis, hypertension, chronic kidney disease, obesity, and type 2 diabetes mellitus (Tang et al., 2017). Interestingly, recent studies have shown that the gut microbiota is closely linked to mechanisms that influence the development of DCM. In this review, the roles of gut microbiota in DCM are discussed and a theoretical basis for the gut microbiota as a new therapeutic target for DCM is provided.

## Gut microbiota and its metabolites

The gut microbiota is a complex microbial community in the gut, consisting of 1,014 species of bacteria, viruses, archaea, fungi, and rotifers (Rajilić-Stojanović et al., 2012; Palm et al., 2015). Most of them belong to *Firmicutes*, *Actinobacteria*, *Bacteroidetes*, *Proteobacteria*, and *Microflora verrucose* families (Koren et al., 2012; Tremaroli and Bäckhed, 2012; Goodrich et al., 2014). Dysregulation of the gut microbiota has been linked to a variety of diseases, such as the metabolic syndrome, atherosclerosis, hypertension, heart failure, chronic kidney disease, obesity, cancer, and diabetes (Bäckhed et al., 2004; Turnbaugh et al., 2006; Jackson and Theiss, 2020). Changes in the composition of gut microbes and their corresponding products, such as lipopolysaccharide (LPS), trimethylamine N-oxide (TMAO), and lactic acid, are associated with risk of diabetes (Yuan et al., 2019). According to Luedde et al. (2017), the microbiome diversity of 20 patients with heart failure and reduced ejection fraction was lower than that of the control group, especially in those with obesity or type 2 diabetes. Reduced diversity of gut microbiota is associated with insulin resistance, dyslipidemia, and inflammatory phenotypes (Le Chatelier et al., 2013). Close attention has been paid to the relationship between cardiovascular diseases (including coronary heart disease, hypertension, and DCM) and gut microbiota in numerous studies. However, findings were inconsistent, gut microbiota has both protective and negative effects on cardiovascular disease. Some studies have shown that the presence of *Enterobacteraceae*, *Ruminococcus gnavus*, and *Eggerthella lenta* increased significantly in the atherosclerosis group compared with the control group, whereas the presence of *butyrate-tensteria nestialis* and *Faecalibacterium prausnitzii* decreased significantly (Jie et al., 2017). In heart failure patients, the level of pathogenic bacteria and *Candida* species (Pasini et al., 2016), increased, and the level of anti-inflammatory bacteria, such as *Faecalibacterium prausnitzii*, *Lact*. *Fermentum*, *Lactobacillus Shirota*, and *F. Prau snitzii* decreased.

Cardiac dysfunction is associated with a variety of changes in microbiota and bacterial metabolite secretion (Bastin and Andreelli, 2020). Some gut bacterial metabolites such as short-chain fatty acids (SCFAs) and trimethylamine (TMA)/TMAO (Brown and Hazen, 2015) also play an important role in cardiovascular disease. However, their role in the heart may be two-sided. On the one hand, TMA plays a role in increasing cardiometabolic risk and is produced from phosphatidylcholine, choline, carnitine, and food through the enzymatic action of the microbiome. TMAO, which has been shown to increase not only the cardiovascular risk, but also the risk of developing cardiac insufficiency, is formed by oxidation of TMA in the liver (Tang et al., 2019). TMAO can induce myocardial hypertrophy and fibrosis in rats with aortic contraction (Li et al., 2019). In addition, TMAO can induce inflammatory responses through SIRT3-SOD2-mtROS (sirtuin-3-superoxide dismutase 2-mitochondrial reactive oxygen species) pathway and nuclear factor κ-light-chain-enhancer of activated B cells (NF-kB) pathway (Seldin et al., 2016; Chen et al., 2017). On the other hand, SCFAs have a protective effect on the heart due to anti-inflammatory properties. This can be explained by different effects of bacteria and metabolites on the host. In case of a maladjusted gut microbiota in an organism, the microbiota may induce a series of changes such as abnormal glucose metabolism, oxidative stress, inflammatory response and apoptosis, all of which are important factors causing DCM. (The roles of these bacteria and their metabolites in oxidative stress, inflammatory response, and other mechanisms is elaborated below) (Table 1).

**TABLE 1:** The influence of different gut microbiota and related products associated with diabetic cardiomyopathy.

	The influence for diabetic cardiomyopathy	
	Positive	Negative
Gut Microbiota	Faecalibacterium prausnitzii	Enterobacteraceae
	Lact. Fermentum	Ruminococcus gnavus
	*Lactobacillus* Shirota	Eggerthella lenta
	Bifidobacterium (BIF)	*candida*
	*Bacteroides fragilis* (*B. fragilis*)	*Vibrio* proteolyticus (VPRH)
Related products associated with Gut Microbiota	Short-chain fatty acid (SCFA)	Trimethylamine (TMA)
	Bile acids (BAs)	Trimethylamine N-oxide (TMAO)
	Butyrate	Branched chain amino acids (BCAA)
	Butyric acid	Lipopolysaccharide (LPS)

**FIGURE 1:**
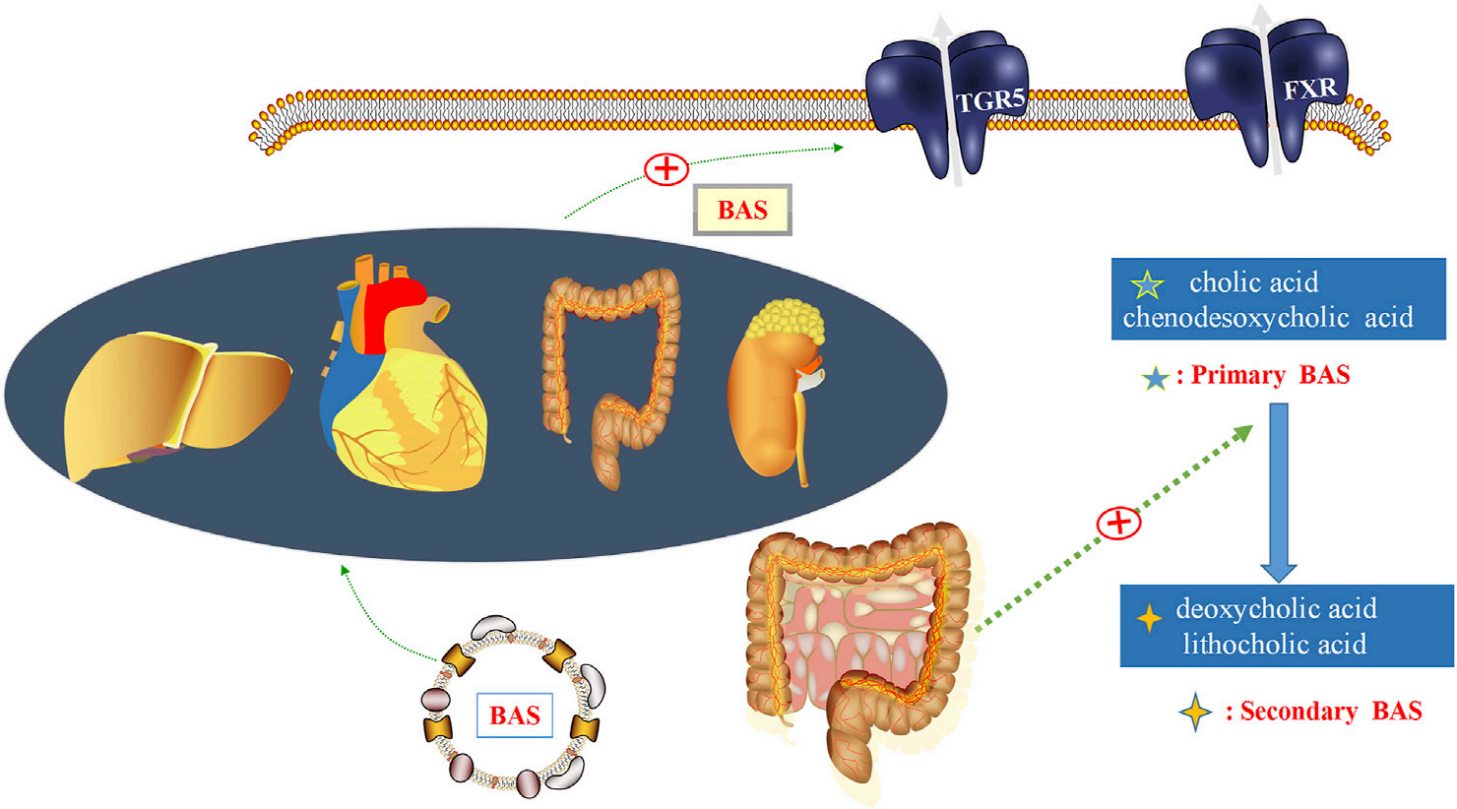
Gut microbiota and bile acids (BAs). In the gut, BAs are a detergent required for the formation of mixed micelles, dissolution, and digestion. BAs regulate metabolic homeostasis by activating BA receptors, such as G protein-coupled bile acid receptor 1 (TGR5), which are expressed in the intestinal tract, heart, liver, kidney, and other organs. Primary bile acids, such as cholic acid and chenodesoxycholic acid, could be converted into secondary BAs, including deoxycholic acid and lithocholic acid, under the regulation of gut microbiota.

G protein-coupled bile acid receptor 1 (TGR5) is a bile acid (BA) specific receptor, which is part of the G protein coupled receptors family. TGR5 is highly expressed in immune cells and gut tissues, as well as in organs such as heart, liver and kidney. TGR5 can be activated by decoupled and coupled BAs (Baars et al., 2015) (Figure 1). BA is an important component of bile. Primary BAs are converted to secondary BAs by microbiota, and changes in the composition of BA pools also affect the distribution of gut microbiota (Sayin et al., 2013). Thus, gut microbiota and its metabolites can influence BA metabolism. The exact role of TGR5 in BA metabolism remains to be clarified. However, circulating BA levels were reduced in TGR5 KO mice compared with WT mice, suggesting that TGR5 plays a role in BA homeostasis (Li et al., 2011). Deng et al. (2019) have confirmed that activation of the TGR5 has a cardioprotective effect against mice myocardial cell damage induced by high glucose. Therefore, BA metabolism may play an important role in linking TGR5 closely to gut microbiota.

## Oxidative stress

### Oxidative stress in DCM

Oxidative stress can induce insulin resistance and β-cell dysfunction, which is a potential culprit in diabetes (Zhang et al., 2020). Oxidative stress has been implicated in the pathogenesis and progression of diabetic vascular complications, including CVD, neuropathy, nephropathy, and retinopathy (Rurali et al., 2013) etc. Studies have shown that DCM increases oxidative stress, and oxidative stress can also accelerate the DCM process (Jia et al., 2018b). In addition, sustained hyperglycemia and the signaling pathway involved in β-oxidation is impaired can lead to reactive oxygen species (ROS) overproduction by disrupting mitochondrial function, increasing mitochondrial oxygen consumption, or activating NOX (an evolutionarily conserved ROS-producing enzyme) (Jia et al., 2016; Zhang and Hu, 2020). Increased ROS levels further induce mitochondrial dysfunction and reduce the oxidative capacity of fatty acids, leading to oxidative stress and inflammation in the heart (Jia et al., 2016). Increased oxidative stress and inflammation in the heart leads to cardiac lipid accumulation, fibrosis, diastolic and systolic dysfunction, and resulting heart failure in patients with diabetes (Jia et al., 2018a).

### Gut microbiota and oxidative stress

It is well known that increased ROS production can induce cardiac mitochondrial dysfunction, and ultimately lead to clinical heart failure in patients with diabetes. Thus, reducing oxidative stress by regulating gut microbiota will be an important mean to treat DCM. The effect of gut microbiota on oxidative stress remains controversial. Recent research shows that physiological levels of oxidative stress can be generated by the gut epithelial lining (Dumitrescu et al., 2018). Gram-negative bacteria could increase lipopolysaccharide (LPS) levels (Lee and Hüttemann, 2014; Mafra et al., 2019), which could produce a large number of ROS, mainly from macrophages and infiltrating neutrophils (Sah et al., 2011). Moreover, Yang and Zhang (2021) proved that TMAO could promote oxidative stress by mediating inositol-requiring enzyme 1α (IRE1α)/X-box binding protein 1 (XBP-1) pathway. However, some researchers have suggested that the gut microbiota can mitigate oxidative stress. For example, a recent report by Kumar et al. (2020) has shown that *Lactobacillus fermentum* (*Lact. fermentum*) significantly attenuated hydrogen peroxide (H_2_O_2_)-induced ROS production in 3T3-L1 preadipocytes. Meanwhile, another study showed that *Lactobacillus Shirota* can protect gut cell-like epithelial cells from 2, 2′-azobis (2-amidinopropane) dihydrochloride-induced oxidative and inflammatory stress by regulating the expression of antioxidant enzymes (Finamore et al., 2018). This contradiction may be explained by differences in the richness and composition of the gut microbiota. Therefore, the most critical issue is to maintain the ecological stability of gut microbiota and improve the types of beneficial bacterias for the host in gut microbiota, which will be a major breakthrough in the treatment of DCM.

## Insulin resistance

### Impaired insulin metabolism and cardiac insulin resistance in DCM

**FIGURE 2:**
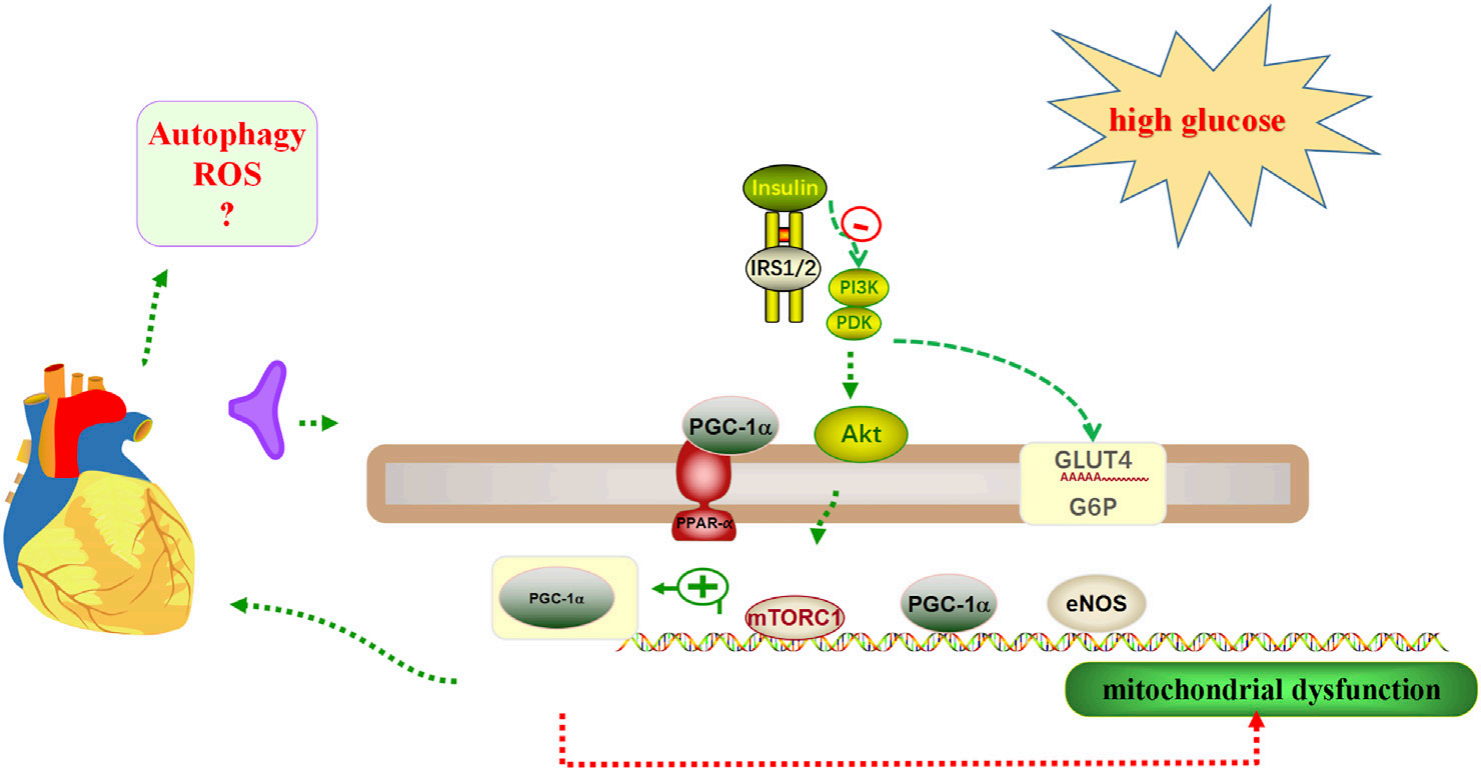
Insulin mechanisms in cardiac glucose regulation. 1) Insulin resistance may occur when cardiomyocytes are exposed to high glucose. 2) The phosphatidylinositol 3-kinase (PI3K)/protein kinase B (Akt) signaling pathway stimulates the translocation of glucose transporter type 4 (GLUT4) to the membrane, thereby resulting in glucose uptake to cells of the heart. However, in a knockout model of the cardiac insulin receptor, cardiac glucose uptake is reduced, resulting in mitochondrial dysfunction, and increased cardiac reactive oxygen species (ROS) production. 3) Mitochondrial dysfunction occurs when the PPARγ coactivator 1α (PGC-1α)/AMP-activated protein kinase (AMPK) signaling pathway is impaired. 4) The level of autophagy may be reduced when the phosphatidylinositol 3-kinase (PI3K)/protein kinase B (Akt)/mammalian target of rapamycin1 (mTORC1) pathway is activated.

Impaired insulin metabolic signaling in the heart plays a key role in the pathogenesis of DCM (Jia et al., 2018b). Cardiac insulin signaling regulates intracellular stability by regulating substrate use, protein synthesis, and cell survival (Jia et al., 2018a). In advanced DCM, the PPARγ coactivator 1α (PGC-1α)/AMP-activated protein kinase (AMPK) signaling pathway involved in β-oxidation is impaired, leading to further mitochondrial dysfunction (Jia et al., 2016). In skeletal muscle, liver, and adipose and heart tissues, glucose transport is mediated by the glucose transporter 4 (GLUT4) (Jia et al., 2016). Under normal physiological conditions, the phosphatidylinositol 3-kinase (PI3K)/protein kinase B (PKB; also called Akt) signaling pathway stimulates the translocation of GLUT4 to the membrane in cardiomyocytes, resulting in glucose uptake of cells in the heart (Jia et al., 2016). In addition, cardiac insulin receptor knockout models showed reduced cardiac glucose uptake, induced mitochondrial dysfunction, and increased cardiac ROS production. In case of dual knockout of insulin receptor substrate-1/2 (IRS-1/2), the ATP content in cardiomyocytes was reduced, cardiomyocyte contractility and function were impaired, and the incidence of fibrosis and heart failure was increased (Bugger et al., 2012; Qi et al., 2013). When the PI3K/protein kinase B (Akt)/mammalian target of rapamycin (mTOR) pathway is activated by insulin signaling, not only protein synthesis is stimulated, but autophagy is also inhibited (Figure 2), which could accelerate the DCM process (Mizushima, 2005; Meijer and Codogno, 2006).

### Gut microbiota and cardiac insulin resistance

A healthy gut microbiota can decrease insulin resistance (Saad et al., 2016). It has been suggested that the response of bacterial SCFAs production levels to nutrient-lipid intake plays a key role in the gut microbiota’s ability to regulate energy balance and metabolism (Kimura et al., 2013; Cani, 2014). Moreover, it was shown that an altered intestinal barrier and a dysregulated gut microbiota cause increased levels of branched chain amino acids (BCAA), secondary Bas, and LPS production, all of which can result in insulin resistance (Saad et al., 2016).

In diet-induced obese mice, supplementation with SCFAs improved insulin resistance and reduced obesity (Perry et al., 2016). In other animal studies, butyric-producing bacteria, such as *F. Prau snitzii*, induces colon L cells to secrete glucagon-like peptide 1 (GLP-1) *via* discrete sampling of the free fatty acid receptor 2(FFAR2), resulting in reduced insulin resistance (Tolhurst et al., 2012; Christiansen et al., 2018). BAs-induced activation of TGR5 promotes the release of GLP-1 by intestinal cells and indirectly affects the secretion of insulin by pancreatic β-cells, thereby affecting insulin sensitivity (Duboc et al., 2014). Therefore, TGR5 may be an important target to offset insulin resistance and reduce damage caused by diabetes.

## Inflammation

**FIGURE 3:**
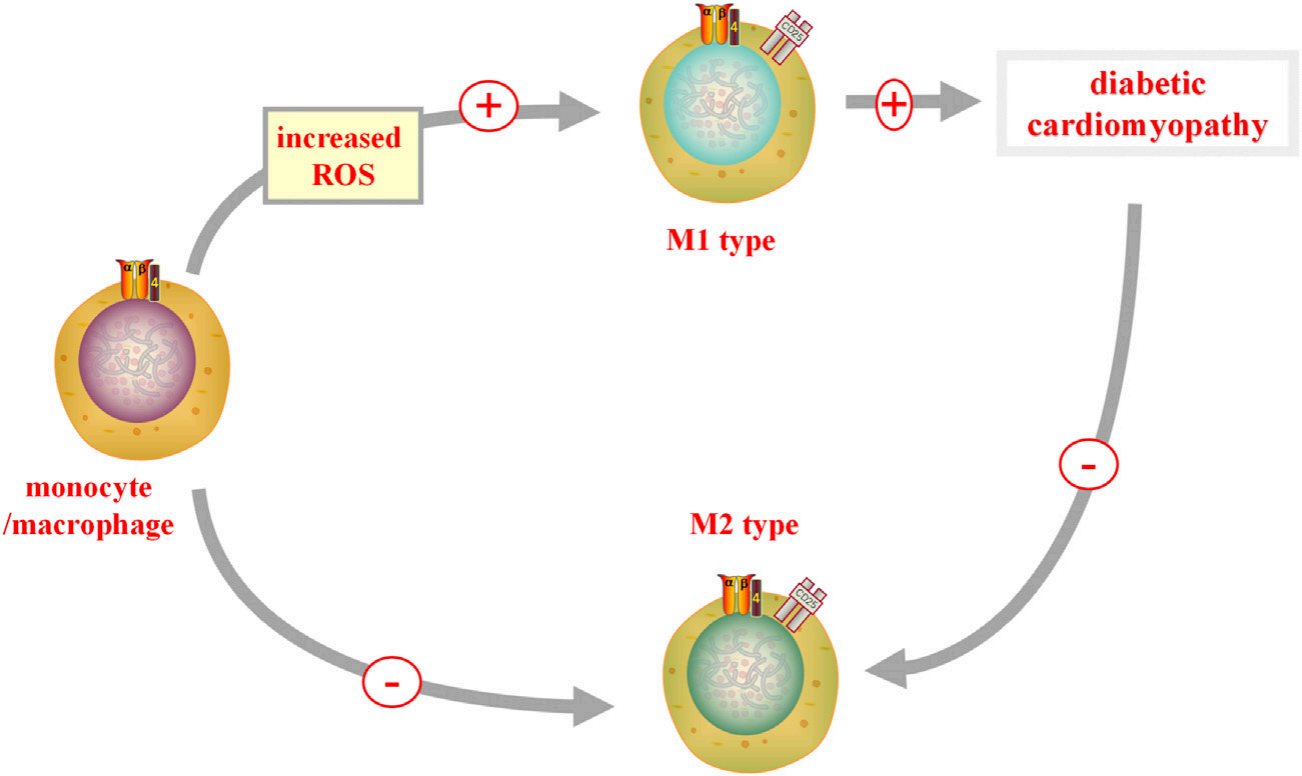
M1 and M2 in DCM. When reactive oxygen species (ROS) is increased, monocytes/macrophages can polarize into a pro-inflammatory M1 phenotype, promoting the occurrence of diabetic cardiomyopathy (DCM). In diabetic heart tissue, the pro-inflammatory polarization of M2 macrophages is inhibited, while the pro-inflammatory polarization of M1 macrophages is upregulated. M1, classically activated macrophages; M2, alternatively activate anti-inflammatory macrophages.

It is generally accepted that an inflammatory response accelerates the development of DCM. The Nucleotide-binding oligomerization domain-like receptor pyrin domains-containing 3 (NLRP3) inflammasome, a new molecular marker of DCM, is activated by impaired insulin metabolic signaling, high FFA levels, and hyperglycemia (Pal et al., 2017). Upon NLRP3 activation, increased migration of monocytes/macrophages through the coronary endothelium occurs, resulting in an increased number of resident cardiac macrophages. When ROS is increased and bioavailable nitric oxide (NO) is reduced, monocytes/macrophages can be polarized into the proinflammatory M1 phenotype (Jia et al., 2016). In a recent study, it was shown that the anti-inflammatory response of M2 macrophages is repressed, whereas the pro-inflammatory polarization of M1 macrophages is upregulated in diabetic heart tissues (Figure 3) (Jia et al., 2015).

Recently, Bartolomaeus et al. (2019) confirmed the anti-inflammatory effects of SCFAs. SCFAs are produced by the fermentation of fibers in the colon and include three main products, namely, propionate, acetate, and butyrate (Chang et al., 2014). Butyrate inhibits proinflammatory factors in gut macrophages, including interleukin-6, interleukin-12, and NO, by inhibition of histone deacetylase (HDAC) (Chang et al., 2014). Besides, propionate has been shown to significantly reduce cardiovascular damage by reducing the number of T-helper 17 cells and effector memory T cells (Bartolomaeus et al., 2019).

However, gut microbiota and its bacterial products not only have anti-inflammatory effects, but also pro-inflammatory effects. For example, Sun et al. (2016) suggested that inflammation induced by TMAO can lead to endothelial dysfunction in human umbilical vein endothelial cells through activation of the inflammasome ROS- thioredoxin interacting protein (TXNIP)-NLRP3. According to Yue et al. (2017), TMAO can activate the release of the inflammatory cytokines interleukin (IL)-18 and IL-1β in the NLRP3 inflammation. TMAO markedly increased inflammatory markers, such as ICAM1, IL-6, E-selectin, and cyclooxygenase-2(COX-2), through activation of the mitogen-activated protein kinase (MAPK) and NF-κB signaling pathways, which then led to vascular inflammation (Seldin et al., 2016). This contradiction may be explained by differences in composition of gut microbiota. It is generally accepted that the inflammatory response is an important pathogenic mechanism of DCM. Together, the data indicate that interfering with the composition of the gut microbiota to increase the number of anti-inflammatory bacteria may result in new ways to treat DCM.

## Autophagy

Autophagy is a highly conserved catabolic process that involves the malformation of proteins, degradation of long-lived proteins, and injury of organelles through the actions of lysosomes (Li et al., 2016). Autophagy occurs in many cells of the cardiovascular system, including vascular smooth muscle cells, myocytes, macrophages, fibroblasts, and endothelial cells (Lavandero et al., 2015). In preclinical trials, autophagy disorders have been observed in diabetic hearts (Kobayashi and Liang, 2015; Jia et al., 2018a). Interestingly, autophagy has two-sided effects. Several investigators have revealed the pathogenic and protective role of autophagy in patients with DCM in type 1 and type 2 diabetes (Dewanjee et al., 2021). This contradiction may be explained by differences in the degree of autophagy. On the one hand, Autophagy is an adaptive protective response of cardiomyocytes to cellular stresses including hyperglycemia, hyperlipidemia, malnutrition, hypoxia, and redox stress (Mellor et al., 2011; Chen et al., 2020). Autophagy also could help restore plasticity in the heart (Lavandero et al., 2015). Besides, autophagy can enhance the antioxidant capacity of cells by activating the nuclear factor erythroid 2-related factor 2 (Nrf2) (Wible and Bratton, 2018). On the other hand, autophagy damage can lead to heart damage (Orogo and Gustafsson Å, 2015). Cardiac dysfunction and abnormalities can cause autophagy injury in diabetic hearts (Xiao et al., 2018; Wu et al., 2020). Autophagy damage by AMP-activated protein kinase (AMPK) suppression can lead to dyslipidemia in the diabetic environment (Zhang et al., 2014), and dyslipidemia can further inhibit cardiac autophagy by enhancing mechanistic target of rapamycin kinase (mTOR) signaling of cardiomyocytes (Glazer et al., 2009). In addition, the myocardial inflammation in diabetic heart can also occur and establish by damaging cardiac autophagy (Zhang et al., 2016). Lavandero et al. (2015) suggested that autophagy hyperactivation may be a cause of heart failure. Autophagy overactivation in the diabetic heart can lead to self-digestion and enhanced ROS production, which are potential contributors to DCM (Xu et al., 2019). Thus, both inhibition and overactivation of cardiac autophagy can have pathological effects on DCM.

Different scholars have different views on the role of gut microbiota in regulating autophagy. Nie et al. (2020) showed that *Bifidobacterium* (BIF) ameliorated tumor necrosis factor alpha (TNF-α)-induced autophagy in colorectal adenocarcinoma cell line (Caco-2) cells by inhibiting p62 levels and expression of autophagy-related markers such as microtubule-associated protein 1 light chain 3- II (LC3II) and Beclin1. Lannucci and colleagues have shown that SCFAs can induce autophagy in hepatocytes through the uncoupling protein 2 (UCP2) (Iannucci et al., 2016). Furthermore, sodium butyrate promoted the decrease of α-synuclein both by inhibiting the autophagy pathway of PI3K/Akt/mTOR and enhancing autophagy-mediated by autophagy-related gene 5 (Atg5) (Qiao et al., 2020). Thus, different bacterias in the gut microbiota have different roles in regulating autophagy.

Combining all, with the emergence of new findings, autophagy has been regarded as a crucial player in regulating DCM. It is well known that gut microbiota can regulate the degree of autophagy through PI3K/Akt/mTOR pathway (Qiao et al., 2020). In addition, PI3K/Akt/mTOR pathway plays an important role in the regulation of autophagy in DCM(Zhao et al., 2020). Therefore, the PI3K/Akt/mTOR pathway may be an important bridge between gut microbiota and DCM in autophagy. In addition, different kinds of bacterias in the gut microbiota also have different effects on autophagy, if we can adjust the gut microbiota to maintain autophagy in a favorable state for the body, it will bring benefits to patients with DCM. However, at present, there is no method to detect the autophagy state in the human heart (Dewanjee et al., 2021). Therefore, in order to find more treatments for DCM, it is very urgent for us to find a way to monitor the exact state of autophagy regulated by bacteria.

## Cell apoptosis and pyroptosis

### Cell apoptosis in DCM

A long-term hyperglycemic state in diabetic patients induces apoptosis by activating caspase apoptosis, which leads to myocardial injury and dysfunction (Wei et al., 2018). It has been shown that long non-coding RNA (lncRNA) can modulate functions in DCM (Yang et al., 2018b; Pant et al., 2018). For example, Zhou et al. (2017) have shown that lncRNA myocardial infarction associated transcript (MIAT) can modulate myocardial cell apoptosis in DCM through microRNA (miR)-22-3p. Also, the modulation of the growth arrest-specific 5(Gas5)/miR-320-3p/transcription factor 3 (Tcf3) pathway in nuclear management coactivator (NMC) and nuclear receptor coactivator (NRC) apoptosis was detected. Moreover, it was demonstrated that Tcf3-activated lncRNA Gas5 modulated the apoptosis of NMC in DCM (Su et al., 2020). However, to date, there is no reported explanation for the low rate of apoptosis in patients with late-stage diabetes and severe cardiac dysfunction (Gu et al., 2018), which needs more experiments to explore it.

### Pyroptosis in DCM

Pyroptosis is defined as programmed cell death associated with inflammation, and characterized by pore formation, cell swelling and destruction of the plasma membrane (Wan et al., 2020). Pyroptosis plays a role in the process of DCM (Yang et al., 2018a). Abnormal pyroptosis of cardiac fibroblasts can induce cardiac dysfunction and collagen deposition, thus aggravating the development of diabetic myocardial fibrosis (Shi et al., 2021). Shi et al. (2021) demonstrated that miR-21–3p can promote myocardial fibroblasts pyroptosis induced by high glucose (HG) *via* enhancing NLRP3 and caspase-1 expression. Recently, another data have shown that the regulation of miRs plays an important role in cell pyroptosis and fibrosis (Li et al., 2019), which bearing out Shi et al. (2021)’s research.

### Gut microbiota affects the development of DCM by regulating apoptosis and pyroptosis

Apoptosis is one of the most studied type of programmed cell death. It is characterized by the formation of unique apoptotic bodies. It is common in patients with heart failure, myocardial infarction and other vascular damage (Zhou et al., 2020). According to the study of Saito et al. (2019), *Bacteroides fragilis* (*B. fragilis*) had a protective effect on the apoptosis of HT29 cells induced by Shiga toxin. However, gut microbiota also has pro-apoptotic effects. Nie et al. (2020) showed that BIF improved TNF-α-induced apoptosis of Caco-2 cells. Li and Elsasser (2005) suggested that butyric acid induced apoptosis and cell cycle arrest in renal epithelial cells.

Pyrodeath, characterized by cell swelling, the release of cytokines, and damage to subcellular organelles, is a type of pro-inflammatory cell death (Liu et al., 2019). Data have shown that TMAO promotes the pyroptosis of vascular endothelial cells through the production of ROS, which leads to the development of atherosclerosis (Cohen et al., 2020). According to the study of Cohen et al. (2020) the gram-negative bacteria *Vibrio proteolyticus* (*VPRH*) from the gut tract of borers induced pyroptosis by activating the NLRP3 inflammasome and caspase-1, resulting in the secretion of IL-1β. By contrast, another study demonstrated that sodium butyrate has an antipyroptosis effect on glomerular endothelial cells and protects them from damage caused by high glucose (Gu et al., 2019).

In summary, gut microbiota has apoptosis, anti-apoptosis, pyroptosis, and anti-pyroptosis effects in host cells. The role of bacterial metabolites of gut microbiota in apoptosis and pyroptosis is still controversial. This controversy may be explained by differences in the composition and species of gut microbiota and its metabolites.

## Gut microbiota and the level of calcium ions

High glucose levels increase Ca^2+^ levels in cardiac myocytes (Cheng et al., 2019). Calcium ions are the key regulator of cardiac hypertrophy; the Ca^2+^-calcineurin-nuclear factor of activated T cells (NFAT) cascade is the main pathway resulting in cardiac hypertrophy (Fiedler and Wollert, 2004). Gut microbiota are the primary source of SCFAs in the plasma (Vinolo et al., 2011). SCFA can regulate the contraction of airway smooth muscle by regulating calcium channels (Mizuta et al., 2020).

**FIGURE 4:**
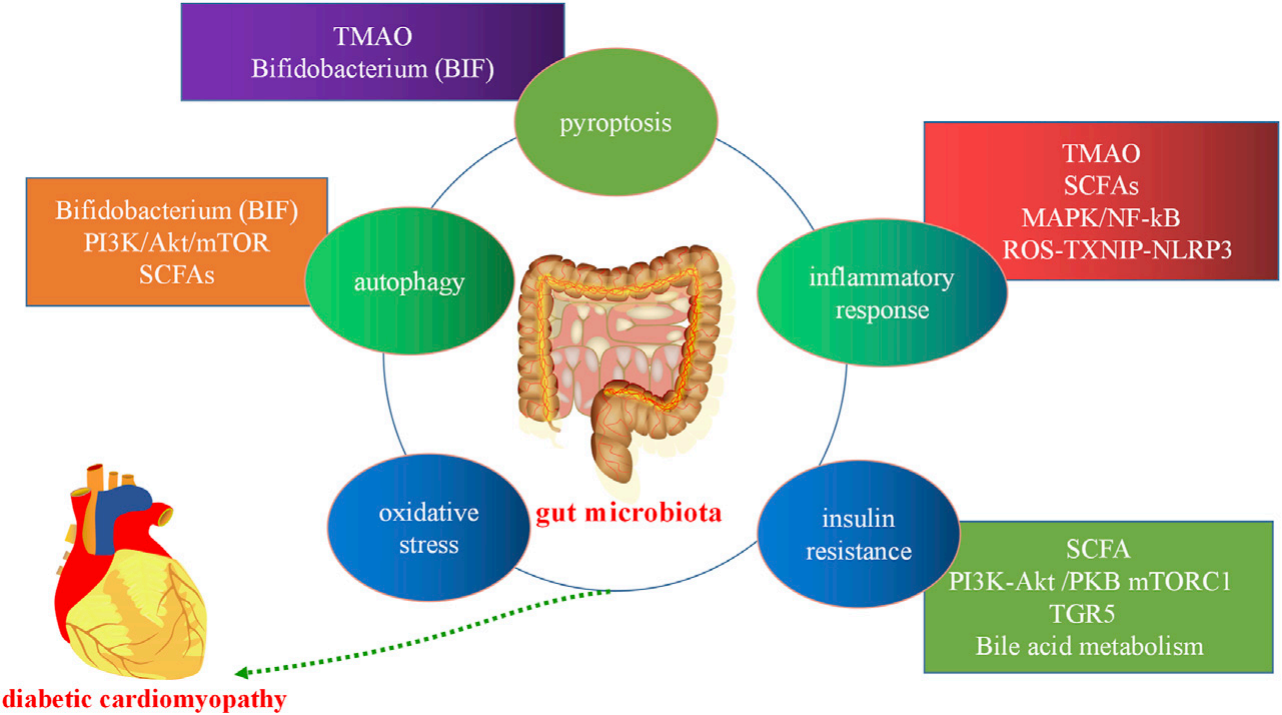
Gut microbiota in diabetic cardiomyopathy. Gut microbiota and its metabolites can affect the development of host cell autophagy, the inflammatory response, oxidative stress, apoptosis, pyroptosis, and insulin resistance through the short-chain fatty acids (SCFAs) metabolic pathway, bile acid (BA) metabolism, and the trimethylamine N-oxide (TMAO) metabolic, mitogen-activated protein kinase (MAPK), and PI3K/Akt/mTOR pathways, among others. PI3K:phosphatidylinositol 3-kinase; Akt, protein kinase B, mTORC: mammalian target of rapamycin.

Gut microbiota and its metabolites can affect the development of pyroptosis, oxidative stress, inflammation, insulin resistance, and autophagy in the host through the regulation TGR5, BA metabolism, and the PI3K/Akt/mTOR, ROS- TXNIP-NLRP3, and MAPK-NF-κB pathways, among others. Therefore, gut microbiota can affect the development of DCM (Figure 4).

## The therapeutic prospect of DCM

The pathogenesis of DCM is various, and it is generally believed that oxidative stress, inflammation, insulin resistance, cell apoptosis and autophagy are closely related to DCM. In recent years, a large number of scholars have found some new methods to prevent and treat DCM by targeting these mechanisms. For example, Gu et al. (2018) have experimently confirmed that inhibition of p53 could prevent DCM by preventing early-stage apoptosis. At present, gut microbiota has been applied to some clinical diseases, such as inflammatory bowel disease (IBD), obesity and some other metabolic diseases (Patterson et al., 2016). The widespread use of probiotics in clinical practice is a good proof. Interestingly, numerous studies have found that the gut microbiota is associated with the pathogenesis of DCM. However, up to now, the role of gut microbiota in DCM is still controversial. Some scholars believe that gut microbiota can reduce myocardial damage in patients with DM by alleviating oxidative stress (Finamore et al., 2018), while others think that gut microbiota can also increase the harmful risk to DCM *via* increasing inflammatory response and insulin resistance (Sun et al., 2016). The contradiction of the two-sided effects of the gut microbiota can mainly be explained by the difference of the bacterial species present in the gut microbiota. It might be possible to reduce oxidative stress, inflammation response, insulin resistance and maintain appropriate levels of autophagy by intervening with the composition of the gut microbiota to increase the species richness of the bacterias which are beneficial for the host in gut microbiota, thereby reducing diabetic myocardial injury. However, it is very difficult for us to intervene with the composition of gut microbiota due to the limited technology available. If we can solve this thorny problem, it will bring a major breakthrough in the treatment of DCM in the future.

Fortunately, there are possibly three ways to modify the composition of the gut microbiota to treat DCM. First, dietary interventions are a good therapeutic option. Experimental studies have shown that dietary modifications for 5 days (short term) can change the number of bacteria in and species of gut microbiota (David et al., 2014). Second, probiotics may be used for the clinical treatment of DCM in the future. Probiotic bacteria mainly derive from the genera *Bifidobacterium* and *Lactobacillus*. Probiotics supplementation has been found to restore the gut microbiota after it had been disrupted. Besides, probiotics supplementation induces changes in the composition of undisrupted gut microbiota. Recent evidence suggests that probiotics affect BA metabolism by altering the microbiota (Baars et al., 2015). Therefore, the probiotics may reduce myocardial injury in DCM by affecting BA metabolism, thereby activating TGR5 expression. Third, fecal microbiota transplantation (FMT) may be a future treatment. FMT is a treatment for patients with gut microecological imbalance. Bacteria or metabolites are introduced from donor feces to the diseased recipient (Cammarota et al., 2014). Bastos et al. (2022) showed that FMT is a safe treatment, they found that FMT prevented weight gain, reduced local TNF-α expression in the ileum and ascending colon, and ameliorated insulin resistance in diabetic mice. The improvement in peripheral insulin sensitivity of male metabolic syndrome recipients after receiving heterogenous gut microbiota from lean donors is attributed to an increased diversity in gut microbiota, including those associated with butyrate production (Vrieze et al., 2012). FMT alone is not sufficient to control glycemic levels effectively. Thus, the regulation of gut microbiota should be combined with other established classical therapies, which can obtain better metabolic parameters (Vallianou et al., 2019; Zhang and Hu, 2020). However, the effectiveness of FMT is challenged by several factors, such as delivery route, number of transplants, fecal volume per sample, disease burden, and target impact (Napolitano and Covasa, 2020). Therefore, FMT is highly difficult to implement and its possibility of success is low. Besides, a major disadvantage of FMT is that viruses are also transplanted (Chehoud et al., 2016) (Table 2). The problem of the balance between the advantages and disadvantages of FMT treatment is still unsolved. In the future, there will be more treatments for DCM by regulating the gut microbiota.

**TABLE 2:** Summary of findings in clinical, cell and animal studies.

Mechanisms and diseases	Animal/Clinical/Cell studies	Summary of findings	References
CVD	Clinical	the CVD risk of cardiomyopathy is 2–5 times higher than in non-diabetic patients	Kannel et al. (1974)
Heart failure	Clinical	The microbiome diversity in those with obesity or type 2 diabetes was lower	Luedde et al. (2017)
Heart failure	Clinical	the level of pathogenic bacteria and *Candida* species increased, the level of anti-inflammatory bacteria decreased	Pasini et al. (2016)
Atherosclerosis	Clinical	Enterobacteraceae, Ruminococcus gnavus, and Eggerthella lenta increased, butyrate-tensteria nestialis and Faecalibacterium prausnitzii decreased	Jie et al. (2017)
CVD	Animal	TMAO can induce myocardial hypertrophy and fibrosis in rats with aortic contraction	Li et al. (2019b)
Inflammation	Animal	TMAO can induce inflammatory responses through SIRT3-SOD2-mtROS pathway and NF-kB pathway	Chen et al. (2017)
		circulating BA levels were reduced in TGR5 KO mice, suggesting that TGR5 plays a role in BA homeostasis	Li et al. (2011)
DCM	Cell	TGR5 has a cardioprotective effect against myocardial cell damage induced by high glucose	Deng et al. (2019)
Oxidative stress	Cell	physiological levels of oxidative stress can be generated by the gut epithelial lining	Dumitrescu et al. (2018)
Autophagy	Cell	PI3K/Akt/mTOR pathway can be significantly attenuated by the exposure of cells to cell-free supernatant of Lact. Fermentum	Kumar et al. (2020)
Insulin resistance	Animal	In diet-induced obese mice, supplementation with SCFAs improved insulin resistance and reduced obesity	Perry et al. (2016)
Insulin resistance	Animal	butyric-producing bacteria reduced insulin resistance	Tolhurst et al. (2012)
Inflammation	Animal	Butyrate inhibits proinflammatory factors in gut macrophages by inhibition of histone deacetylase	Chang et al. (2014)
Inflammation	Cell	inflammation induced by TMAO can lead to endothelial dysfunction in human umbilical vein endothelial cells	Sun et al. (2016)
Inflammation	Cell	TMAO can activate the release of the inflammatory cytokines IL-18 and IL-1β	Yue et al. (2017)
Autophagy	Animal	Cardiac dysfunction and abnormalities can cause autophagy injury in diabetic hearts	Xiao et al., 2018; Wu et al., 2020
Autophagy	Animal	Autophagy damage by AMPK suppression can lead to dyslipidemia in the diabetic environment	Zhang et al. (2014)
Autophagy	Cell	BIF improved TNF-α-induced autophagy in Caco-2 cells by inhibiting p62 levels and expression of autophagy-related markers	Nie et al. (2020)
Autophagy	Cell	SCFAs can induce autophagy in hepatocytes through the UCP2	Iannucci et al. (2016)
Autophagy	Cell	sodium butyrate promoted the decrease of α-synuclein by regulating the autophagy pathway	Qiao et al. (2020)
Cell apoptosis	Cell	A long-term hyperglycemic state induces apoptosis by activating caspase apoptosis, which leads to myocardial injury and dysfunction	Wei et al. (2018)
Cell apoptosis	Animal	lncRNA MIAT can modulate myocardial cell apoptosis in DCM through miR-22-3p	Zhou et al. (2017)
Pyroptosis	Animal and Cell	Abnormal pyroptosis of cardiac fibroblasts can induce cardiac dysfunction and collagen deposition, thus aggravating the development of diabetic myocardial fibrosis	Shi et al. (2021)
Cell apoptosis	Animal and Cell	*Bacteroides fragilis* (*B. fragilis*) had a protective effect on the apoptosis of HT29 cells induced by Shiga toxin	Saito et al. (2019)
Pyroptosis	Animal and Cell	TMAO promotes the pyroptosis of vascular endothelial cells through the production of ROS, which leads to the development of atherosclerosis	Cohen et al. (2020)
Pyroptosis	Cell	sodium butyrate has an antipyroptosis effect on glomerular endothelial cells and protects them from damage caused by high glucose	Gu et al. (2019)
Oxidative stress	Cell	gut microbiota can reduce myocardial damage by alleviating oxidative stress	Finamore et al. (2018)
Insulin resistance	Animal	FMT prevented weight gain, reduced local TNF-α expression in the ileum and ascending colon, and ameliorated insulin resistance in diabetic mice	Bastos et al. (2022)
Insulin resistance	Cell	The improvement in peripheral insulin sensitivity of male metabolic syndrome recipients after receiving heterogenous gut microbiota from lean donors is attributed to an increased diversity in gut microbiota	Vrieze et al. (2012)
FMT	Clinical	a major disadvantage of FMT is that viruses are also transplanted	Chehoud et al. (2016)

Abbreviations: CVD, cardiovascular diseases; SIRT3-SOD2-mtROS, sirtuin-3-superoxide dismutase 2-mitochondrial reactive oxygen species; TMAO, trimethylamineN-oxide; NF-kB, nuclear factor κ-light-chain-enhancer of activated B cells; DCM, diabetic cardiomyopathy; TGR5, G protein-coupled bile acid receptor 1; PI3K, phosphatidylinositol 3-kinase; Akt, protein kinase B; mTOR, mammalian target of rapamycin; SCFAs, short-chain fatty acids; IL, interleukin; AMPK, AMP-activated protein kinase; BIF, Bifidobacterium; UCP2, uncoupling protein 2; MIAT, myocardial infarction associated transcript; lncRNA, long non-coding RNA; ROS, reactive oxygen species; miR, microRNA; FMT, fecal microbiota transplantation.

## Conclusion

DCM has a serious impact on people’s quality of life, and even threatens lives of patients. Therefore, it is very important to find new therapeutic targets to treat DCM. The pathogenesis of DCM is complex and diverse. It is generally accepted that the mechanisms include oxidative stress, inflammation, insulin resistance, and cell apoptosis. In recent years, it has been suggested that the development of DCM is closely related to autophagy and cell pyroptosis.

The gut microbiota has become topic of interest in research. Some studies have shown that gut microbiota plays an important role in cardiovascular disease. However, the role of gut microbiota in DCM may be two-sided. On the one side, some bacteria can reduce myocardial damage by reducing the inflammatory response, while others can aggravate myocardial damage by increasing the oxidative stress response. This contradiction can mainly be explained by the difference in the composition of gut microbiota in patients. Therefore, finding an effective way to intervene with the composition of gut microbiota and regulate the metabolism of gut microbes will be a major breakthrough in the clinical treatment of DCM.

## References

[B1] Baars A. Oosting A. Knol J. Garssen J. Van Bergenhenegouwen J. (2015). The gut microbiota as a therapeutic target in IBD and metabolic disease: A role for the bile acid receptors FXR and TGR5. Microorganisms 3, 641–666. 10.3390/microorganisms3040641 PubMed Abstract | 10.3390/microorganisms3040641 | Google Scholar 27682110 PMC5023267

[B2] Bäckhed F. Ding H. Wang T. Hooper L. V. Koh G. Y. Nagy A. (2004). The gut microbiota as an environmental factor that regulates fat storage. Proc. Natl. Acad. Sci. U. S. A. 101, 15718–15723. 10.1073/pnas.0407076101 PubMed Abstract | 10.1073/pnas.0407076101 | Google Scholar 15505215 PMC524219

[B3] Bartolomaeus H. Balogh A. Yakoub M. Homann S. Markó L. Höges S. (2019). Short-chain fatty acid propionate protects from hypertensive cardiovascular damage. Circulation 139, 1407–1421. 10.1161/circulationaha.118.036652 PubMed Abstract | 10.1161/circulationaha.118.036652 | Google Scholar 30586752 PMC6416008

[B4] Bastin M. Andreelli F. (2020). The gut microbiota and diabetic cardiomyopathy in humans. Diabetes Metab. 46, 197–202. 10.1016/j.diabet.2019.10.003 PubMed Abstract | 10.1016/j.diabet.2019.10.003 | Google Scholar 31678397

[B5] Bastos R. M. C. Simplício-Filho A. Sávio-Silva C. Oliveira L. F. V. Cruz G. N. F. Sousa E. H. (2022). Fecal microbiota transplant in a pre-clinical model of type 2 diabetes mellitus, obesity and diabetic kidney disease. Int. J. Mol. Sci. 23, 3842. 10.3390/ijms23073842 PubMed Abstract | 10.3390/ijms23073842 | Google Scholar 35409202 PMC8998923

[B6] Brown J. M. Hazen S. L. (2015). The gut microbial endocrine organ: bacterially derived signals driving cardiometabolic diseases. Annu. Rev. Med. 66, 343–359. 10.1146/annurev-med-060513-093205 PubMed Abstract | 10.1146/annurev-med-060513-093205 | Google Scholar 25587655 PMC4456003

[B7] Bugger H. Riehle C. Jaishy B. Wende A. R. Tuinei J. Chen D. (2012). Genetic loss of insulin receptors worsens cardiac efficiency in diabetes. J. Mol. Cell. Cardiol. 52, 1019–1026. 10.1016/j.yjmcc.2012.02.001 PubMed Abstract | 10.1016/j.yjmcc.2012.02.001 | Google Scholar 22342406 PMC3327790

[B8] Cammarota G. Ianiro G. Gasbarrini A. (2014). Fecal microbiota transplantation for the treatment of *Clostridium difficile* infection: a systematic review. J. Clin. Gastroenterol. 48, 693–702. 10.1097/mcg.0000000000000046 PubMed Abstract | 10.1097/mcg.0000000000000046 | Google Scholar 24440934

[B9] Cani P. D. (2014). Metabolism in 2013: The gut microbiota manages host metabolism. Nat. Rev. Endocrinol. 10, 74–76. 10.1038/nrendo.2013.240 PubMed Abstract | 10.1038/nrendo.2013.240 | Google Scholar 24322652

[B10] Chang P. V. Hao L. Offermanns S. Medzhitov R. (2014). The microbial metabolite butyrate regulates intestinal macrophage function via histone deacetylase inhibition. Proc. Natl. Acad. Sci. U. S. A. 111, 2247–2252. 10.1073/pnas.1322269111 PubMed Abstract | 10.1073/pnas.1322269111 | Google Scholar 24390544 PMC3926023

[B11] Chehoud C. Dryga A. Hwang Y. Nagy-Szakal D. Hollister E. B. Luna R. A. (2016). Transfer of viral communities between human individuals during fecal microbiota transplantation. mBio 7, e00322. 10.1128/mBio.00322-16 PubMed Abstract | 10.1128/mBio.00322-16 | Google Scholar 27025251 PMC4817255

[B12] Chen M. L. Zhu X. H. Ran L. Lang H. D. Yi L. Mi M. T. (2017). Trimethylamine-N-Oxide induces vascular inflammation by activating the NLRP3 inflammasome through the SIRT3-SOD2-mtROS signaling pathway. J. Am. Heart Assoc. 6, e006347. 10.1161/jaha.117.006347 PubMed Abstract | 10.1161/jaha.117.006347 | Google Scholar 28871042 PMC5634285

[B13] Chen Y. Hua Y. Li X. Arslan I. M. Zhang W. Meng G. (2020). Distinct types of cell death and the implication in diabetic cardiomyopathy. Front. Pharmacol. 11, 42. 10.3389/fphar.2020.00042 PubMed Abstract | 10.3389/fphar.2020.00042 | Google Scholar 32116717 PMC7018666

[B14] Cheng K. C. Chang W. T. Kuo F. Y. Chen Z. C. Li Y. Cheng J. T. (2019). TGR5 activation ameliorates hyperglycemia-induced cardiac hypertrophy in H9c2 cells. Sci. Rep. 9, 3633. 10.1038/s41598-019-40002-0 PubMed Abstract | 10.1038/s41598-019-40002-0 | Google Scholar 30842472 PMC6403401

[B15] Christiansen C. B. Gabe M. B. N. Svendsen B. Dragsted L. O. Rosenkilde M. M. Holst J. J. (2018). The impact of short-chain fatty acids on GLP-1 and PYY secretion from the isolated perfused rat colon. Am. J. Physiol. Gastrointest. Liver Physiol. 315, G53–g65. 10.1152/ajpgi.00346.2017 PubMed Abstract | 10.1152/ajpgi.00346.2017 | Google Scholar 29494208

[B16] Cohen H. Baram N. Edry-Botzer L. Munitz A. Salomon D. Gerlic M. (2020). Vibrio pore-forming leukocidin activates pyroptotic cell death via the NLRP3 inflammasome. Emerg. Microbes Infect. 9, 278–290. 10.1080/22221751.2020.1720526 PubMed Abstract | 10.1080/22221751.2020.1720526 | Google Scholar 32013758 PMC7034064

[B17] David L. A. Maurice C. F. Carmody R. N. Gootenberg D. B. Button J. E. Wolfe B. E. (2014). Diet rapidly and reproducibly alters the human gut microbiome. Nature 505, 559–563. 10.1038/nature12820 PubMed Abstract | 10.1038/nature12820 | Google Scholar 24336217 PMC3957428

[B18] Deng L. Chen X. Zhong Y. Wen X. Cai Y. Li J. (2019). Activation of TGR5 partially alleviates high glucose-induced cardiomyocyte injury by inhibition of inflammatory responses and oxidative stress. Oxid. Med. Cell. Longev. 2019, 6372786. 10.1155/2019/6372786 PubMed Abstract | 10.1155/2019/6372786 | Google Scholar 31871553 PMC6906824

[B19] Dewanjee S. Vallamkondu J. Kalra R. S. John A. Reddy P. H. Kandimalla R. (2021). Autophagy in the diabetic heart: A potential pharmacotherapeutic target in diabetic cardiomyopathy. Ageing Res. Rev. 68, 101338. 10.1016/j.arr.2021.101338 PubMed Abstract | 10.1016/j.arr.2021.101338 | Google Scholar 33838320

[B20] Dillmann W. H. (2019). Diabetic cardiomyopathy. Circ. Res. 124, 1160–1162. 10.1161/circresaha.118.314665 PubMed Abstract | 10.1161/circresaha.118.314665 | Google Scholar 30973809 PMC6578576

[B21] Duboc H. Taché Y. Hofmann A. F. (2014). The bile acid TGR5 membrane receptor: from basic research to clinical application. Dig. Liver Dis. 46, 302–312. 10.1016/j.dld.2013.10.021 PubMed Abstract | 10.1016/j.dld.2013.10.021 | Google Scholar 24411485 PMC5953190

[B22] Dumitrescu L. Popescu-Olaru I. Cozma L. Tulbă D. Hinescu M. E. Ceafalan L. C. (2018). Oxidative stress and the microbiota-gut-brain Axis. Oxid. Med. Cell. Longev. 2018, 2406594. 10.1155/2018/2406594 PubMed Abstract | 10.1155/2018/2406594 | Google Scholar 30622664 PMC6304899

[B23] Fiedler B. Wollert K. C. (2004). Interference of antihypertrophic molecules and signaling pathways with the Ca2+-calcineurin-NFAT cascade in cardiac myocytes. Cardiovasc. Res. 63, 450–457. 10.1016/j.cardiores.2004.04.002 PubMed Abstract | 10.1016/j.cardiores.2004.04.002 | Google Scholar 15276470

[B24] Finamore A. Ambra R. Nobili F. Garaguso I. Raguzzini A. Serafini M. (2018). Redox role of Lactobacillus casei Shirota against the cellular damage induced by 2, 2'-azobis (2-amidinopropane) dihydrochloride-induced oxidative and inflammatory stress in enterocytes-like epithelial cells. Front. Immunol. 9, 1131. 10.3389/fimmu.2018.01131 PubMed Abstract | 10.3389/fimmu.2018.01131 | Google Scholar 29881384 PMC5976738

[B25] Glazer H. P. Osipov R. M. Clements R. T. Sellke F. W. Bianchi C. (2009). Hypercholesterolemia is associated with hyperactive cardiac mTORC1 and mTORC2 signaling. Cell Cycle 8, 1738–1746. 10.4161/cc.8.11.8619 PubMed Abstract | 10.4161/cc.8.11.8619 | Google Scholar 19395857 PMC3308015

[B26] Goodrich J. K. Waters J. L. Poole A. C. Sutter J. L. Koren O. Blekhman R. (2014). Human genetics shape the gut microbiome. Cell 159, 789–799. 10.1016/j.cell.2014.09.053 PubMed Abstract | 10.1016/j.cell.2014.09.053 | Google Scholar 25417156 PMC4255478

[B27] Gu J. Huang W. Zhang W. Zhao T. Gao C. Gan W. (2019). Sodium butyrate alleviates high-glucose-induced renal glomerular endothelial cells damage via inhibiting pyroptosis. Int. Immunopharmacol. 75, 105832. 10.1016/j.intimp.2019.105832 PubMed Abstract | 10.1016/j.intimp.2019.105832 | Google Scholar 31473434

[B28] Gu J. Wang S. Guo H. Tan Y. Liang Y. Feng A. (2018). Inhibition of p53 prevents diabetic cardiomyopathy by preventing early-stage apoptosis and cell senescence, reduced glycolysis, and impaired angiogenesis. Cell Death Dis. 9, 82. 10.1038/s41419-017-0093-5 PubMed Abstract | 10.1038/s41419-017-0093-5 | Google Scholar 29362483 PMC5833384

[B29] Iannucci L. F. Sun J. Singh B. K. Zhou J. Kaddai V. A. Lanni A. (2016). Short chain fatty acids induce UCP2-mediated autophagy in hepatic cells. Biochem. Biophys. Res. Commun. 480, 461–467. 10.1016/j.bbrc.2016.10.072 PubMed Abstract | 10.1016/j.bbrc.2016.10.072 | Google Scholar 27773823

[B30] Jackson D. N. Theiss A. L. (2020). Gut bacteria signaling to mitochondria in intestinal inflammation and cancer. Gut Microbes 11, 285–304. 10.1080/19490976.2019.1592421 PubMed Abstract | 10.1080/19490976.2019.1592421 | Google Scholar 30913966 PMC7524274

[B31] Jia G. Demarco V. G. Sowers J. R. (2016). Insulin resistance and hyperinsulinaemia in diabetic cardiomyopathy. Nat. Rev. Endocrinol. 12, 144–153. 10.1038/nrendo.2015.216 PubMed Abstract | 10.1038/nrendo.2015.216 | Google Scholar 26678809 PMC4753054

[B32] Jia G. Habibi J. Demarco V. G. Martinez-Lemus L. A. Ma L. Whaley-Connell A. T. (2015). Endothelial mineralocorticoid receptor deletion prevents diet-induced cardiac diastolic dysfunction in females. Hypertension 66, 1159–1167. 10.1161/hypertensionaha.115.06015 PubMed Abstract | 10.1161/hypertensionaha.115.06015 | Google Scholar 26441470 PMC4644106

[B33] Jia G. Hill M. A. Sowers J. R. (2018a). Diabetic cardiomyopathy: An update of mechanisms contributing to this clinical entity. Circ. Res. 122, 624–638. 10.1161/circresaha.117.311586 PubMed Abstract | 10.1161/circresaha.117.311586 | Google Scholar 29449364 PMC5819359

[B34] Jia G. Whaley-Connell A. Sowers J. R. (2018b). Diabetic cardiomyopathy: a hyperglycaemia- and insulin-resistance-induced heart disease. Diabetologia 61, 21–28. 10.1007/s00125-017-4390-4 PubMed Abstract | 10.1007/s00125-017-4390-4 | Google Scholar 28776083 PMC5720913

[B35] Jie Z. Xia H. Zhong S. L. Feng Q. Li S. Liang S. (2017). The gut microbiome in atherosclerotic cardiovascular disease. Nat. Commun. 8, 845. 10.1038/s41467-017-00900-1 PubMed Abstract | 10.1038/s41467-017-00900-1 | Google Scholar 29018189 PMC5635030

[B36] Kannel W. B. Hjortland M. Castelli W. P. (1974). Role of diabetes in congestive heart failure: the framingham study. Am. J. Cardiol. 34, 29–34. 10.1016/0002-9149(74)90089-7 PubMed Abstract | 10.1016/0002-9149(74)90089-7 | Google Scholar 4835750

[B37] Kimura I. Ozawa K. Inoue D. Imamura T. Kimura K. Maeda T. (2013). The gut microbiota suppresses insulin-mediated fat accumulation via the short-chain fatty acid receptor GPR43. Nat. Commun. 4, 1829. 10.1038/ncomms2852 PubMed Abstract | 10.1038/ncomms2852 | Google Scholar 23652017 PMC3674247

[B38] Kobayashi S. Liang Q. (2015). Autophagy and mitophagy in diabetic cardiomyopathy. Biochim. Biophys. Acta 1852, 252–261. 10.1016/j.bbadis.2014.05.020 PubMed Abstract | 10.1016/j.bbadis.2014.05.020 | Google Scholar 24882754

[B39] Koren O. Goodrich J. K. Cullender T. C. Spor A. Laitinen K. Bäckhed H. K. (2012). Host remodeling of the gut microbiome and metabolic changes during pregnancy. Cell 150, 470–480. 10.1016/j.cell.2012.07.008 PubMed Abstract | 10.1016/j.cell.2012.07.008 | Google Scholar 22863002 PMC3505857

[B40] Kumar R. Sharma A. Gupta M. Padwad Y. Sharma R. (2020). Cell-free culture supernatant of probiotic Lactobacillus fermentum protects against H(2)O(2)-induced premature senescence by suppressing ROS-akt-mTOR Axis in murine preadipocytes. Probiotics Antimicrob. Proteins 12, 563–576. 10.1007/s12602-019-09576-z PubMed Abstract | 10.1007/s12602-019-09576-z | Google Scholar 31332650

[B41] Lavandero S. Chiong M. Rothermel B. A. Hill J. A. (2015). Autophagy in cardiovascular biology. J. Clin. Invest. 125, 55–64. 10.1172/jci73943 PubMed Abstract | 10.1172/jci73943 | Google Scholar 25654551 PMC4382263

[B42] Le Chatelier E. Nielsen T. Qin J. Prifti E. Hildebrand F. Falony G. (2013). Richness of human gut microbiome correlates with metabolic markers. Nature 500, 541–546. 10.1038/nature12506 PubMed Abstract | 10.1038/nature12506 | Google Scholar 23985870

[B43] Lee I. Hüttemann M. (2014). Energy crisis: the role of oxidative phosphorylation in acute inflammation and sepsis. Biochim. Biophys. Acta 1842, 1579–1586. 10.1016/j.bbadis.2014.05.031 PubMed Abstract | 10.1016/j.bbadis.2014.05.031 | Google Scholar 24905734 PMC4147665

[B44] Li C. J. Elsasser T. H. (2005). Butyrate-induced apoptosis and cell cycle arrest in bovine kidney epithelial cells: involvement of caspase and proteasome pathways. J. Anim. Sci. 83, 89–97. 10.2527/2005.83189x PubMed Abstract | 10.2527/2005.83189x | Google Scholar 15583047

[B45] Li J. Xue J. Wang D. Dai X. Sun Q. Xiao T. (2019a). Regulation of gasdermin D by miR-379-5p is involved in arsenite-induced activation of hepatic stellate cells and in fibrosis via secretion of IL-1β from human hepatic cells. Metallomics. 11, 483–495. 10.1039/c8mt00321a PubMed Abstract | 10.1039/c8mt00321a | Google Scholar 30643918

[B46] Li L. Xu J. He L. Peng L. Zhong Q. Chen L. (2016). The role of autophagy in cardiac hypertrophy. Acta Biochim. Biophys. Sin. 48, 491–500. 10.1093/abbs/gmw025 PubMed Abstract | 10.1093/abbs/gmw025 | Google Scholar 27084518 PMC4913516

[B47] Li T. Holmstrom S. R. Kir S. Umetani M. Schmidt D. R. Kliewer S. A. (2011). The G protein-coupled bile acid receptor, TGR5, stimulates gallbladder filling. Mol. Endocrinol. 25, 1066–1071. 10.1210/me.2010-0460 PubMed Abstract | 10.1210/me.2010-0460 | Google Scholar 21454404 PMC3100601

[B48] Li Z. Wu Z. Yan J. Liu H. Liu Q. Deng Y. (2019b). Gut microbe-derived metabolite trimethylamine N-oxide induces cardiac hypertrophy and fibrosis. Lab. Invest. 99, 346–357. 10.1038/s41374-018-0091-y PubMed Abstract | 10.1038/s41374-018-0091-y | Google Scholar 30068915

[B49] Liu J. Wang Y. Meng H. Yu J. Lu H. Li W. (2019). Butyrate rather than LPS subverts gingival epithelial homeostasis by downregulation of intercellular junctions and triggering pyroptosis. J. Clin. Periodontol. 46, 894–907. 10.1111/jcpe.13162 PubMed Abstract | 10.1111/jcpe.13162 | Google Scholar 31241781

[B50] Luedde M. Winkler T. Heinsen F. A. Rühlemann M. C. Spehlmann M. E. Bajrovic A. (2017). Heart failure is associated with depletion of core intestinal microbiota. ESC Heart Fail. 4, 282–290. 10.1002/ehf2.12155 PubMed Abstract | 10.1002/ehf2.12155 | Google Scholar 28772054 PMC5542738

[B51] Mafra D. Borges N. A. Lindholm B. Stenvinkel P. (2019). Mitochondrial dysfunction and gut microbiota imbalance: An intriguing relationship in chronic kidney disease. Mitochondrion 47, 206–209. 10.1016/j.mito.2018.11.006 PubMed Abstract | 10.1016/j.mito.2018.11.006 | Google Scholar 30408595

[B52] Meijer A. J. Codogno P. (2006). Signalling and autophagy regulation in health, aging and disease. Mol. Asp. Med. 27, 411–425. 10.1016/j.mam.2006.08.002 PubMed Abstract | 10.1016/j.mam.2006.08.002 | Google Scholar 16973212

[B53] Mellor K. M. Bell J. R. Young M. J. Ritchie R. H. Delbridge L. M. (2011). Myocardial autophagy activation and suppressed survival signaling is associated with insulin resistance in fructose-fed mice. J. Mol. Cell. Cardiol. 50, 1035–1043. 10.1016/j.yjmcc.2011.03.002 PubMed Abstract | 10.1016/j.yjmcc.2011.03.002 | Google Scholar 21385586

[B54] Mizushima N. (2005). The pleiotropic role of autophagy: from protein metabolism to bactericide. Cell Death Differ. 12, 1535–1541. 10.1038/sj.cdd.4401728 PubMed Abstract | 10.1038/sj.cdd.4401728 | Google Scholar 16247501

[B55] Mizuta K. Sasaki H. Zhang Y. Matoba A. Emala C. W. Sr. (2020). The short-chain free fatty acid receptor FFAR3 is expressed and potentiates contraction in human airway smooth muscle. Am. J. Physiol. Lung Cell. Mol. Physiol. 318, L1248–l1260. 10.1152/ajplung.00357.2019 PubMed Abstract | 10.1152/ajplung.00357.2019 | Google Scholar 32209026 PMC7347267

[B56] Napolitano M. Covasa M. (2020). Microbiota transplant in the treatment of obesity and diabetes: Current and future perspectives. Front. Microbiol. 11, 590370. 10.3389/fmicb.2020.590370 PubMed Abstract | 10.3389/fmicb.2020.590370 | Google Scholar 33304339 PMC7693552

[B57] Nie N. Bai C. Song S. Zhang Y. Wang B. Li Z. (2020). Bifidobacterium plays a protective role in TNF-α-induced inflammatory response in Caco-2 cell through NF-κB and p38MAPK pathways. Mol. Cell. Biochem. 464, 83–91. 10.1007/s11010-019-03651-3 PubMed Abstract | 10.1007/s11010-019-03651-3 | Google Scholar 31741130

[B58] Orogo A. M. Gustafsson Å B. (2015). Therapeutic targeting of autophagy: potential and concerns in treating cardiovascular disease. Circ. Res. 116, 489–503. 10.1161/circresaha.116.303791 PubMed Abstract | 10.1161/circresaha.116.303791 | Google Scholar 25634972 PMC4313578

[B59] Pal P. B. Sonowal H. Shukla K. Srivastava S. K. Ramana K. V. (2017). Aldose reductase mediates NLRP3 inflammasome-initiated innate immune response in hyperglycemia-induced Thp1 monocytes and male mice. Endocrinology 158, 3661–3675. 10.1210/en.2017-00294 PubMed Abstract | 10.1210/en.2017-00294 | Google Scholar 28938395 PMC5659696

[B60] Palm N. W. De Zoete M. R. Flavell R. A. (2015). Immune-microbiota interactions in health and disease. Clin. Immunol. 159, 122–127. 10.1016/j.clim.2015.05.014 PubMed Abstract | 10.1016/j.clim.2015.05.014 | Google Scholar 26141651 PMC4943041

[B61] Pant T. Dhanasekaran A. Fang J. Bai X. Bosnjak Z. J. Liang M. (2018). Current status and strategies of long noncoding RNA research for diabetic cardiomyopathy. BMC Cardiovasc. Disord. 18, 197. 10.1186/s12872-018-0939-5 PubMed Abstract | 10.1186/s12872-018-0939-5 | Google Scholar 30342478 PMC6196023

[B62] Pasini E. Aquilani R. Testa C. Baiardi P. Angioletti S. Boschi F. (2016). Pathogenic gut flora in patients with chronic heart failure. JACC. Heart Fail. 4, 220–227. 10.1016/j.jchf.2015.10.009 PubMed Abstract | 10.1016/j.jchf.2015.10.009 | Google Scholar 26682791

[B63] Patterson E. Ryan P. M. Cryan J. F. Dinan T. G. Ross R. P. Fitzgerald G. F. (2016). Gut microbiota, obesity and diabetes. Postgrad. Med. J. 92, 286–300. 10.1136/postgradmedj-2015-133285 PubMed Abstract | 10.1136/postgradmedj-2015-133285 | Google Scholar 26912499

[B64] Perry R. J. Peng L. Barry N. A. Cline G. W. Zhang D. Cardone R. L. (2016). Acetate mediates a microbiome-brain-β-cell axis to promote metabolic syndrome. Nature 534, 213–217. 10.1038/nature18309 PubMed Abstract | 10.1038/nature18309 | Google Scholar 27279214 PMC4922538

[B65] Qi Y. Xu Z. Zhu Q. Thomas C. Kumar R. Feng H. (2013). Myocardial loss of IRS1 and IRS2 causes heart failure and is controlled by p38α MAPK during insulin resistance. Diabetes 62, 3887–3900. 10.2337/db13-0095 PubMed Abstract | 10.2337/db13-0095 | Google Scholar 24159000 PMC3806607

[B66] Qiao C. M. Sun M. F. Jia X. B. Shi Y. Zhang B. P. Zhou Z. L. (2020). Sodium butyrate causes α-synuclein degradation by an Atg5-dependent and PI3K/Akt/mTOR-related autophagy pathway. Exp. Cell Res. 387, 111772. 10.1016/j.yexcr.2019.111772 PubMed Abstract | 10.1016/j.yexcr.2019.111772 | Google Scholar 31836471

[B67] Rajilić-Stojanović M. Heilig H. G. Tims S. Zoetendal E. G. De Vos W. M. (2012). Long-term monitoring of the human intestinal microbiota composition. Environ. Microbiol. 15, 1146–1159. 10.1111/1462-2920.12023 10.1111/1462-2920.12023 | Google Scholar 23286720

[B68] Rurali E. Noris M. Chianca A. Donadelli R. Banterla F. Galbusera M. (2013). ADAMTS13 predicts renal and cardiovascular events in type 2 diabetic patients and response to therapy. Diabetes 62, 3599–3609. 10.2337/db13-0530 PubMed Abstract | 10.2337/db13-0530 | Google Scholar 23733198 PMC3781447

[B69] Saad M. J. Santos A. Prada P. O. (2016). Linking gut microbiota and inflammation to obesity and insulin resistance. Physiol. (Bethesda) 31, 283–293. 10.1152/physiol.00041.2015 PubMed Abstract | 10.1152/physiol.00041.2015 | Google Scholar 27252163

[B70] Sah S. P. Tirkey N. Kuhad A. Chopra K. (2011). Effect of quercetin on lipopolysaccharide induced-sickness behavior and oxidative stress in rats. Indian J. Pharmacol. 43, 192–196. 10.4103/0253-7613.77365 PubMed Abstract | 10.4103/0253-7613.77365 | Google Scholar 21572657 PMC3081461

[B71] Saito K. Suzuki R. Koyanagi Y. Isogai H. Yoneyama H. Isogai E. (2019). Inhibition of enterohemorrhagic *Escherichia coli* O157:H7 infection in a gnotobiotic mouse model with pre-colonization by Bacteroides strains. Biomed. Rep. 10, 175–182. 10.3892/br.2019.1193 PubMed Abstract | 10.3892/br.2019.1193 | Google Scholar 30906546 PMC6403472

[B72] Sayin S. I. Wahlström A. Felin J. Jäntti S. Marschall H. U. Bamberg K. (2013). Gut microbiota regulates bile acid metabolism by reducing the levels of tauro-beta-muricholic acid, a naturally occurring FXR antagonist. Cell Metab. 17, 225–235. 10.1016/j.cmet.2013.01.003 PubMed Abstract | 10.1016/j.cmet.2013.01.003 | Google Scholar 23395169

[B73] Seldin M. M. Meng Y. Qi H. Zhu W. Wang Z. Hazen S. L. (2016). Trimethylamine N-oxide promotes vascular inflammation through signaling of mitogen-activated protein kinase and nuclear factor-κb. J. Am. Heart Assoc. 5, e002767. 10.1161/jaha.115.002767 PubMed Abstract | 10.1161/jaha.115.002767 | Google Scholar 26903003 PMC4802459

[B74] Shi P. Zhao X. D. Shi K. H. Ding X. S. Tao H. (2021). MiR-21-3p triggers cardiac fibroblasts pyroptosis in diabetic cardiac fibrosis via inhibiting androgen receptor. Exp. Cell Res. 399, 112464. 10.1016/j.yexcr.2020.112464 PubMed Abstract | 10.1016/j.yexcr.2020.112464 | Google Scholar 33385416

[B75] Su D. Ju Y. Han W. Yang Y. Wang F. Wang T. (2020). Tcf3‐activated lncRNA Gas5 regulates newborn mouse cardiomyocyte apoptosis in diabetic cardiomyopathy. J. Cell. Biochem. 121, 4337–4346. 10.1002/jcb.29630 PubMed Abstract | 10.1002/jcb.29630 | Google Scholar 32003049

[B76] Sun X. Jiao X. Ma Y. Liu Y. Zhang L. He Y. (2016). Trimethylamine N-oxide induces inflammation and endothelial dysfunction in human umbilical vein endothelial cells via activating ROS-TXNIP-NLRP3 inflammasome. Biochem. Biophys. Res. Commun. 481, 63–70. 10.1016/j.bbrc.2016.11.017 PubMed Abstract | 10.1016/j.bbrc.2016.11.017 | Google Scholar 27833015

[B77] Tang S.-G. Liu X.-Y. Wang S.-P. Wang H.-H. Jovanović A. Tan W. (2019a). Trimetazidine prevents diabetic cardiomyopathy by inhibiting Nox2/TRPC3-induced oxidative stress. J. Pharmacol. Sci. 139, 311–318. 10.1016/j.jphs.2019.01.016 PubMed Abstract | 10.1016/j.jphs.2019.01.016 | Google Scholar 30962089

[B78] Tang W. H. Kitai T. Hazen S. L. (2017). Gut microbiota in cardiovascular health and disease. Circ. Res. 120, 1183–1196. 10.1161/circresaha.117.309715 PubMed Abstract | 10.1161/circresaha.117.309715 | Google Scholar 28360349 PMC5390330

[B79] Tang W. H. W. Li D. Y. Hazen S. L. (2019b). Dietary metabolism, the gut microbiome, and heart failure. Nat. Rev. Cardiol. 16, 137–154. 10.1038/s41569-018-0108-7 PubMed Abstract | 10.1038/s41569-018-0108-7 | Google Scholar 30410105 PMC6377322

[B80] Tolhurst G. Heffron H. Lam Y. S. Parker H. E. Habib A. M. Diakogiannaki E. (2012). Short-chain fatty acids stimulate glucagon-like peptide-1 secretion via the G-protein-coupled receptor FFAR2. Diabetes 61, 364–371. 10.2337/db11-1019 PubMed Abstract | 10.2337/db11-1019 | Google Scholar 22190648 PMC3266401

[B81] Tremaroli V. Bäckhed F. (2012). Functional interactions between the gut microbiota and host metabolism. Nature 489, 242–249. 10.1038/nature11552 PubMed Abstract | 10.1038/nature11552 | Google Scholar 22972297

[B82] Turnbaugh P. J. Ley R. E. Mahowald M. A. Magrini V. Mardis E. R. Gordon J. I. (2006). An obesity-associated gut microbiome with increased capacity for energy harvest. Nature 444, 1027–1031. 10.1038/nature05414 PubMed Abstract | 10.1038/nature05414 | Google Scholar 17183312

[B83] Vallianou N. G. Stratigou T. Tsagarakis S. (2019). Metformin and gut microbiota: their interactions and their impact on diabetes. Horm. (Athens) 18, 141–144. 10.1007/s42000-019-00093-w PubMed Abstract | 10.1007/s42000-019-00093-w | Google Scholar 30719628

[B84] Vinolo M. A. Rodrigues H. G. Nachbar R. T. Curi R. (2011). Regulation of inflammation by short chain fatty acids. Nutrients 3, 858–876. 10.3390/nu3100858 PubMed Abstract | 10.3390/nu3100858 | Google Scholar 22254083 PMC3257741

[B85] Vrieze A. Van Nood E. Holleman F. Salojärvi J. Kootte R. S. Bartelsman J. F. (2012). Transfer of intestinal microbiota from lean donors increases insulin sensitivity in individuals with metabolic syndrome. Gastroenterology 143, 913–916. 10.1053/j.gastro.2012.06.031 PubMed Abstract | 10.1053/j.gastro.2012.06.031 | Google Scholar 22728514

[B86] Wan P. Su W. Zhang Y. Li Z. Deng C. Li J. (2020). LncRNA H19 initiates microglial pyroptosis and neuronal death in retinal ischemia/reperfusion injury. Cell Death Differ. 27, 176–191. 10.1038/s41418-019-0351-4 PubMed Abstract | 10.1038/s41418-019-0351-4 | Google Scholar 31127201 PMC7206022

[B87] Wei X. Yang Y. Jiang Y. J. Lei J. M. Guo J. W. Xiao H. (2018). Relaxin ameliorates high glucose-induced cardiomyocyte hypertrophy and apoptosis via the Notch1 pathway. Exp. Ther. Med. 15, 691–698. 10.3892/etm.2017.5448 PubMed Abstract | 10.3892/etm.2017.5448 | Google Scholar 29399073 PMC5772593

[B88] Wible D. J. Bratton S. B. (2018). Reciprocity in ROS and autophagic signaling. Curr. Opin. Toxicol. 7, 28–36. 10.1016/j.cotox.2017.10.006 PubMed Abstract | 10.1016/j.cotox.2017.10.006 | Google Scholar 29457143 PMC5810588

[B89] Wu Q. Q. Liu C. Cai Z. Xie Q. Hu T. Duan M. (2020). High-mobility group AT-hook 1 promotes cardiac dysfunction in diabetic cardiomyopathy via autophagy inhibition. Cell Death Dis. 11, 160. 10.1038/s41419-020-2316-4 PubMed Abstract | 10.1038/s41419-020-2316-4 | Google Scholar 32123163 PMC7052237

[B90] Xiao Y. Wu Q. Q. Duan M. X. Liu C. Yuan Y. Yang Z. (2018). TAX1BP1 overexpression attenuates cardiac dysfunction and remodeling in STZ-induced diabetic cardiomyopathy in mice by regulating autophagy. Biochim. Biophys. Acta. Mol. Basis Dis. 1864, 1728–1743. 10.1016/j.bbadis.2018.02.012 PubMed Abstract | 10.1016/j.bbadis.2018.02.012 | Google Scholar 29476905

[B91] Xu T. Ding W. Ji X. Ao X. Liu Y. Yu W. (2019). Oxidative stress in cell death and cardiovascular diseases. Oxid. Med. Cell. Longev. 2019, 9030563. 10.1155/2019/9030563 PubMed Abstract | 10.1155/2019/9030563 | Google Scholar 31781356 PMC6875219

[B92] Yang F. Qin Y. Lv J. Wang Y. Che H. Chen X. (2018a). Silencing long non-coding RNA Kcnq1ot1 alleviates pyroptosis and fibrosis in diabetic cardiomyopathy. Cell Death Dis. 9, 1000. 10.1038/s41419-018-1029-4 PubMed Abstract | 10.1038/s41419-018-1029-4 | Google Scholar 30250027 PMC6155223

[B93] Yang F. Qin Y. Wang Y. Li A. Lv J. Sun X. (2018b). LncRNA KCNQ1OT1 mediates pyroptosis in diabetic cardiomyopathy. Cell. Physiol. Biochem. 50, 1230–1244. 10.1159/000494576 PubMed Abstract | 10.1159/000494576 | Google Scholar 30355944

[B94] Yang G. Zhang X. (2021). TMAO promotes apoptosis and oxidative stress of pancreatic acinar cells by mediating IRE1α-XBP-1 pathway. Saudi J. Gastroenterol. 27, 361–369. 10.4103/sjg.sjg_12_21 PubMed Abstract | 10.4103/sjg.sjg_12_21 | Google Scholar 34755714 PMC8656330

[B95] Yuan T. Yang T. Chen H. Fu D. Hu Y. Wang J. (2019). New insights into oxidative stress and inflammation during diabetes mellitus-accelerated atherosclerosis. Redox Biol. 20, 247–260. 10.1016/j.redox.2018.09.025 PubMed Abstract | 10.1016/j.redox.2018.09.025 | Google Scholar 30384259 PMC6205410

[B96] Yue C. Yang X. Li J. Chen X. Zhao X. Chen Y. (2017). Trimethylamine N-oxide prime NLRP3 inflammasome via inhibiting ATG16L1-induced autophagy in colonic epithelial cells. Biochem. Biophys. Res. Commun. 490, 541–551. 10.1016/j.bbrc.2017.06.075 PubMed Abstract | 10.1016/j.bbrc.2017.06.075 | Google Scholar 28629999

[B97] Zhang J. Cheng Y. Gu J. Wang S. Zhou S. Wang Y. (2016). Fenofibrate increases cardiac autophagy via FGF21/SIRT1 and prevents fibrosis and inflammation in the hearts of Type 1 diabetic mice. Clin. Sci. 130, 625–641. 10.1042/cs20150623 PubMed Abstract | 10.1042/cs20150623 | Google Scholar 26795437

[B98] Zhang L. Ding W. Y. Wang Z. H. Tang M. X. Wang F. Li Y. (2016). Early administration of trimetazidine attenuates diabetic cardiomyopathy in rats by alleviating fibrosis, reducing apoptosis and enhancing autophagy. J. Transl. Med. 14, 109. 10.1186/s12967-016-0849-1 PubMed Abstract | 10.1186/s12967-016-0849-1 | Google Scholar 27121077 PMC4848862

[B99] Zhang P. Li T. Wu X. Nice E. C. Huang C. Zhang Y. (2020). Oxidative stress and diabetes: antioxidative strategies. Front. Med. 14, 583–600. 10.1007/s11684-019-0729-1 PubMed Abstract | 10.1007/s11684-019-0729-1 | Google Scholar 32248333

[B100] Zhang Q. Hu N. (2020). Effects of metformin on the gut microbiota in obesity and type 2 diabetes mellitus. Diabetes Metab. Syndr. Obes. 13, 5003–5014. 10.2147/dmso.S286430 PubMed Abstract | 10.2147/dmso.S286430 | Google Scholar 33364804 PMC7751595

[B101] Zhang Z. Wang S. Zhou S. Yan X. Wang Y. Chen J. (2014). Sulforaphane prevents the development of cardiomyopathy in type 2 diabetic mice probably by reversing oxidative stress-induced inhibition of LKB1/AMPK pathway. J. Mol. Cell. Cardiol. 77, 42–52. 10.1016/j.yjmcc.2014.09.022 PubMed Abstract | 10.1016/j.yjmcc.2014.09.022 | Google Scholar 25268649

[B102] Zhao G. Zhang X. Wang H. Chen Z. (2020). Beta carotene protects H9c2 cardiomyocytes from advanced glycation end product-induced endoplasmic reticulum stress, apoptosis, and autophagy via the PI3K/Akt/mTOR signaling pathway. Ann. Transl. Med. 8, 647. 10.21037/atm-20-3768 PubMed Abstract | 10.21037/atm-20-3768 | Google Scholar 32566584 PMC7290636

[B103] Zhou W. Cheng Y. Zhu P. Nasser M. I. Zhang X. Zhao M. (2020). Implication of gut microbiota in cardiovascular diseases. Oxid. Med. Cell. Longev. 2020, 5394096. 10.1155/2020/5394096 PubMed Abstract | 10.1155/2020/5394096 | Google Scholar 33062141 PMC7533754

[B104] Zhou X. Zhang W. Jin M. Chen J. Xu W. Kong X. (2017). lncRNA MIAT functions as a competing endogenous RNA to upregulate DAPK2 by sponging miR-22-3p in diabetic cardiomyopathy. Cell Death Dis. 8, e2929. 10.1038/cddis.2017.321 PubMed Abstract | 10.1038/cddis.2017.321 | Google Scholar 28703801 PMC5550866

